# Pleistocene climate change promoted rapid diversification of aquatic invertebrates in Southeast Australia

**DOI:** 10.1186/1471-2148-12-142

**Published:** 2012-08-09

**Authors:** Oliver Hawlitschek, Lars Hendrich, Marianne Espeland, Emmanuel FA Toussaint, Martin J Genner, Michael Balke

**Affiliations:** 1 Zoologische Staatssammlung, Münchhausenstr. 21, Munich, 81247, Germany; 2 Department of Organismic and Evolutionary Biology, Harvard University, 26 Oxford Street, Cambridge, MA, 02138, USA; 3 School of Biological Sciences, University of Bristol, Woodland Road, Bristol, BS8 1UG, UK; 4 GeoBioCenter, Ludwig-Maximilians-Universität, Richard-Wagner-Str. 10, Munich, 80333, Germany

## Abstract

**Background:**

The Pleistocene Ice Ages were the most recent geohistorical event of major global impact, but their consequences for most parts of the Southern hemisphere remain poorly known. We investigate a radiation of ten species of *Sternopriscus*, the most species-rich genus of epigean Australian diving beetles. These species are distinct based on genital morphology but cannot be distinguished readily by mtDNA and nDNA because of genotype sharing caused by incomplete lineage sorting. Their genetic similarity suggests a Pleistocene origin.

**Results:**

We use a dataset of 3858 bp of mitochondrial and nuclear DNA to reconstruct a phylogeny of *Sternopriscus* using gene and species trees. Diversification analyses support the finding of a recent rapid speciation event with estimated speciation rates of up to 2.40 species per MY, which is considerably higher than the proposed average rate of 0.16 species per MY for insects. Additionally, we use ecological niche modeling and analyze data on habitat preferences to test for niche divergence between species of the recent *Sternopriscus* radiation. These analyses show that the species can be characterized by a set of ecological variables referring to habitat, climate and altitude.

**Conclusions:**

Our results suggest that the repeated isolation of populations in glacial refugia might have led to divergent ecological adaptations and the fixation of morphological traits supporting reproductive isolation and therefore may have promoted speciation. The recent *Sternopriscus* radiation fulfills many characteristics of a species flock and would be the first described example of an aquatic insect species flock. We argue that the species of this group may represent a stage in speciation past the species flock condition because of their mostly broad and often non-overlapping ranges and preferences for different habitat types.

## Background

Global biodiversity is shaped by the processes of speciation and extinction, whose rates vary depending on region, environment, taxonomic group and geohistorical events [1-3]. Evidence for shifts in the rates of speciation and extinction have been inferred from the fossil record since early paleontology [4], and advances in molecular biology have greatly improved our capabilities to study these processes particularly for taxa with sparse or inconsistent fossil evidence [5,6].

The most recent geohistorical event of major global impact on biodiversity was the Pleistocene glaciations, or Ice Ages, which represent the largest expansion of cold climates since the Permian period 250 million years (MY) earlier. Until 10,000 years ago, temperatures repeatedly oscillated between warm and cold phases. The effects on the environment varied depending on geographical region, but were always accompanied by major biotic shifts. Boreal regions, particularly in the Northern hemisphere, were mostly glaciated and drove species into refugia [7]. In the tropics and subtropics, where glaciations were mostly restricted to high altitudes, a similar effect was attributed to the aridification of formerly humid forest habitats [8]. It has been a matter of discussion whether these cycles of environmental change promoted speciation [9] or whether species responded solely by shifting their ranges toward ecologically suitable areas [10]. In Australia, glaciations occurred only at its highest elevations, but biota faced an ongoing process of aridification that was initiated in the Miocene *c*. 15 million years ago (MYA) when Australia drifted northward [11]. During the Ice Ages, the relatively rapid shifts between warm and wet *versus* cold and dry conditions had severe consequences particularly for the fauna [12,13]. Aquatic environments were strongly affected by oscillations between arid and humid conditions [14].

The genesis of the Australian arid zone promoted radiations in various organism groups, e.g., hypogean faunas in the ground waters underneath the spreading deserts, which most likely began with the onset of the aridification *c.* 15 MYA [14]. However, many rapid radiations of insects dating back only 2 MY or less have been described from all around the world. Coyne & Orr [15] proposed an average speciation rate of 0.16 species per MY, which is exceeded by an order of magnitude by the fastest known radiation [16-18]. Phylogenies of such young radiations based on mitochondrial gene trees are often poorly resolved, and species may appear para- or polyphyletic because of shared alleles with other species, which may be the result of incomplete lineage sorting or hybridization [19]. Species trees may cope with these problems: in a method based on a coalescent model and Bayesian inference, all gene trees are co-estimated and embedded in a single species tree whose tips represent species and not single samples [20,21].

Aside from morphological and molecular characters, ecological factors can be useful to distinguish and even delimit species. Many studies have shown that a variety of climate factors often have a profound effect on the distributions of species, and these factors can be combined to project potential distributions of species in an Ecological Niche Modeling (ENM) approach [22,23]. The predictive powers of this method have been demonstrated [24], and it has been successfully applied in species delimitations [22,25]. Naturally, the distinction of species based on differences in their responses to ecological factors is sensible only if there are actual response differences. Evidence of niche conservatism in closely related species, promoting allopatric speciation, is abundant [26]. However, in many examples of rapid radiations in limited geographic areas niche divergence appears to be the more common condition, and closely related species show different responses to ecological factors [2004, 27].

The focus of our study is on the genus *Sternopriscus* (Coleoptera: Dytiscidae: Hydroporini), which is the most species-rich epigean genus of Australian diving beetles and contains 28 species [27,28]. *Sternopriscus* species inhabit a wide variety of lentic and lotic habitats from sea level to high altitudes. 18 species are found in southeastern Australia, of which four species are endemic to Tasmania. The corresponding freshwater ecoregions according to Abell *et al.*[29] are Eastern Coastal Australia, Bass Strait Drainages, Southern Tasmania, and small parts of the Murray-Darling region. Unlike many other aquatic invertebrates, such as crustaceans and gastropods, most species of epigean aquatic beetles use flight to colonize new habitats. Therefore, the presence of suitable habitats most likely has a higher impact on aquatic beetle distribution than the drainage systems defining the biogeographic regions of Abell *et al.*[29]. Nevertheless, only 2 of these 18 species have a wider distribution over mainland Australia (*S*. *multimaculatus* and *S. clavatus*). 6 species, including some taxonomically and geographically isolated species, are endemic to peaty habitats in the southwest, in an area with cold and humid climate during winter, and 5 species are distributed over the tropical north, including one endemic species in the deep gorges of the Pilbara. None, or only one, species is shared by 2 or more of these areas of endemism. This distribution reflects the restriction of all but the widespread pioneer species *S*. *multimaculatus* to the more humid coastal areas of Australia. The high level of endemism in the southeast and southwest suggests that the arid barrier between these two regions is long-standing. Another strong pattern is the virtual absence of *S*. *tarsalis* group members from the north and southwest regions of the continent, whereas members of the *S*. *hansardii* group, with highly modified male antennae and median lobes, are more widespread [27,28].

Based on male morphological characters, the genus has been divided into 3 groups: the *S. hansardii* group (11 species), the *S. tarsalis* group (13 species), and 4 ‘phylogenetically isolated’ species. The species in the *S. tarsalis* group have been assigned to 3 species complexes: the *S. tarsalis* complex (2 species), the *S. meadfootii* complex (5), and the *S. tasmanicus* complex (3). 3 species have not been assigned to any complex. The 10 species belonging to the *S. tarsalis*, *S. meadfootii* and *S. tasmanicus* complexes in the *S. tarsalis* group are genetically similar and centered in mesic southeastern Australia. Below, we refer to this group of species as the *S. tarsalis* radiation (STR). The STR is supposedly the result of recent diversification; some of these morphologically well-defined species occur in sympatry, and some in syntopy [27,28,30]. Previous genetic studies [30] suggest that species belonging to the STR are not easily delimited using mtDNA and nDNA.

In this study, we attempt to test the following hypotheses: (1) the delimitation of species in the STR, based on morphological characters, can be supported by genetic or ecological data; (2) the STR species originated in a rapid and recent diversification event, most likely in the Pleistocene; and (3) the radiation of the STR was promoted by the Pleistocene climate oscillations. We use a molecular phylogeny with gene and species trees and diversification rate analyses to investigate how environmental change has affected speciation and extinction rates in the genus *Sternopriscus*. We then discuss which factors might have promoted lineage diversification in the STR and whether the molecular similarities are caused by hybridization or incomplete lineage sorting. Aside from the results of our molecular phylogeny, we use phylogeographic network analyses and ENM paired with empirical ecological data in an attempt to reveal how this diversification was promoted.

## Methods

### Sampling and laboratory procedures

Specimens were collected by sweeping aquatic dip nets and metal kitchen strainers in shallow water or operating black-light traps [27] and preserved in 96% ethanol. DNA was extracted non-destructively using Qiagen blood and tissue kits (Qiagen, Hilden). Primers are listed in Additional file 1: Table S1. New sequences were submitted to GenBank under accession numbers [EMBL:HE818935] to [EMBL:HE819178]; *cox1* data are [EMBL:FR732513] to [EMBL:FR733591]. The individual beetles from which we extracted and sequenced DNA each bear a green cardboard label that indicates our DNA extraction number (e.g., “DNA 1780 M. Balke”). This number links the DNA sample, the dry mounted voucher specimen and the GenBank entries.

### Phylogenetic analyses

The aligned 3858 bp dataset contains three mitochondrial (16 S rRNA, cytochrome oxidase b (*cob*), and cytochrome *c* oxidase subunit I (*cox1*)) and four nuclear gene fragments (18 S rRNA, arginine kinase (ARK), histone 3 (h3), and histone 4 (h4)) for 54 specimens of 25 *Sternopriscus* species and 2 Hydroporini outgroups, *Barretthydrus stepheni* and *Carabhydrus niger*. Among the known species of *Sternopriscus*, only *S. mouchampsi* and *S. pilbaraensis* were not available for sequencing. *S. emmae* was excluded from the phylogenetic analyses because we only had DNA from museum specimens and only obtained a short *cox1* sequence. DNA alignment was performed in MUSCLE 3.7 [31]. We then used jModelTest 0.1.1 [32] to identify appropriate substitution models for each gene separately, assessing lnL, AIC and BIC results and giving preference to BIC. To evaluate different partition schemes, we performed a Bayes factor test with MrBayes 3.1 [33] and Tracer v1.5 [34]. The eleven schemes tested were mitochondrial *versus* nuclear, protein-coding *versus* ribosomal, and according to codon positions (1 + 2 *versus* 3 or one partition for each codon position). We used raxmlGUI 0.93 [35] for maximum likelihood analyses with 1000 fast bootstrap repeats. MrBayes 3.1 [33] was used for Bayesian analyses, with two runs and four chains with 30,000,000 generations (samplefreq = 1,000 and 25% burnin). Runs were checked for convergence and normal distribution in Tracer v1.5 [34]. We then used parsimony analysis to infer phylogenetic relations as implemented in the program TNT v1.1, which we also used to run 500 jackknife replications (removal 36%) to assess node stability [36] (hit the best tree 5 times, keep 10,000 trees in memory). Finally, we used coalescent-based species tree inference models in *BEAST v1.6.1 [21] for comparison with the results of the phylogenetic gene tree. *BEAST requires *a‐priori* designation of species, which we performed based on morphological data [27,28]. We conducted two runs over 100,000,000 generations (sample freq = 1,000 and 20% burnin) and checked for convergence and normal distribution in Tracer v1.5 [34]. Additionally, as proposed in Pepper *et al.*[13], we repeated this analysis using simpler substitution models (HKY + G). All analyses in MUSCLE and MrBayes were run on the CIPRES Portal 2.2 [37]. Pairwise distances were calculated in MEGA 5.0 [38].

### Lineage diversification and radiation

Analyses were conducted in R with the packages APE [39] and Laser [40]. Based on the phylogenetic tree created in MrBayes, we used the ‘chronopl’ function of APE to create an ultrametric tree in R and cropped all representatives but one of each species. We then constructed Lineage-Through-Time (LTT) plots [41] and calculated γ-statistics [42]. Because new species continue to be discovered in Australia and incomplete taxon sampling might influence γ-statistics, we conducted a Monte Carlo constant rates (mccr) test with 10,000 replicates, assuming 10% missing species. We then tested the fit of two rate-constant [41] and four rate-variable diversification models [43] to our dataset. Finally, we calculated *p*-values by simulating 10,000 trees with original numbers of present and missing species for a pure-birth scenario and for various birth-death rates (b = 0.5 and d = 0.0, 0.25, 0.5, 0.75 and 0.9). To be able to understand the effect of the near-tip radiation in the STR, we also tested γ for a tree in which this group was treated as a single taxon.

Because of a lack of reliable calibration points, we cannot rely on molecular clock analyses to estimate node ages in the *Sternopriscus* phylogeny. However, we attempt to approximate the age of the rapid radiation in the STR using the standard mutation rates of the *cox1* gene [44,45]. We apply the equation presented in Mendelson & Shaw [16] to estimate the relative speed of this radiation for comparison with other known rapid radiations in insects. For young and monophyletic radiations, such as the STR, the equation is = lnN/t, where is the rate of diversification, N is the number of extant species, and t is the divergence time.

### Phylogeographic structure analysis

We assembled a matrix of 710 bp of only *cox1* for 79 specimens of STR species to investigate the phylogeographic structure of this group. Additional sequences were obtained from Hendrich *et al.*[30]. The standard population genetic statistics Fu's Fs [46] and Tajima's D [47] were calculated, and mismatch distribution analyses to untangle demographic histories were performed using DnaSP 5.10 [48]. The multiple sequences were collapsed in haplotypes also using DnaSP 5.10. A minimum-spanning network was then inferred in Arlequin 3.5.1.3 [49] and used to create a minimum-spanning tree (MST) using Hapstar 0.5 [50]. The scalable vector graphics editor Inkscape 0.48 was further used to map geographic and taxonomic information on the MST.

### Distinguishing incomplete lineage sorting from hybridization

We used an approach developed by Joly *et al.*[51], and employed in Joyce *et al.*[52] and Genner & Turner [53] to test whether the haplotype sharing between STR species was mainly the result of incomplete lineage sorting or influenced by hybridization. In this approach, mtDNA evolution is simulated using a species tree topology that assumes hybridization is absent. If low genetic distances between species pairs are due to incomplete lineage sorting, these similarly low genetic distances should be observed in the simulations. If low genetic distances between species pairs are due to hybridization, then significantly lower genetic distances should be present than observed in the simulations. First, we ran another *BEAST [21] analysis of a subset of the entire multilocus dataset containing only the STR species, using the HKY + G model for 11,000,000 generations (samplefreq = 1,000 and 10% burnin). Second, we used MrModeltest [54] to estimate the parameters of the substitution model for the *cox1* dataset from Hendrich *et al.*[30], which was previously used in the phylogeographic structure analysis. Third, we conducted a run of the JML software [55] using the same *cox1* dataset, the locus rate of *cox1* as yielded by *BEAST, a heredity scalar of 0.5, and the parameters yielded by MrModeltest.

### Ecological niche modeling and analyses

In an attempt to detect possible divergence in response to climatic variables in their ranges, we created ecological niche models (ENMs) for the species of the STR. We excluded *S. montanus* and *S. williamsi* from the ENM analyses because of an insufficient number of localities. Our models were based on a total of 215 distribution points [27,28] (Additional file 2: Table S2) and unpublished data by L. Hendrich. With the exception of three records of *S. wehnckei*, all STR species occur in broad sympatry in southeastern Australia including Tasmania.

We preliminarily selected climate variables according to ecological requirements considered critical for the species. Bioclimatic variables [56] represent either annual means or maxima and minima in temperature and precipitation, or variables correlating temperature and precipitation, e.g., "mean temperature of wettest quarter" (BIO8). Such variables are useful for representing the seasonality of habitats [25]. After the preliminary selection, we used the ENMtools software [57] to calculate correlations between the selected climate layers in the area of interest. In our final selection, we removed layers until no two layers had correlation coefficients (r²) higher than 0.75. ENMs for each species were created in Maxent 3.3.2 [58] (our procedure: Hawlitschek *et al.*[25]). Suitable background areas that were reachable by the species were defined by drawing minimum convex polygons around the species records, as suggested by Phillips *et al.*[59]. We conducted runs with 25% test percentage, 100 bootstrap repeats, jackknifing to measure variable importance and logistic output format. Model validation was performed by calculating the area under the curve (AUC) [60]. To compare ENMs of different *Sternopriscus* species, we measured niche overlap [57] in ENMtools. We also used ENMtools' niche identity test [61] with 500 repeats because the niche overlap values alone do not allow any statements whether the ENMs generated for the two species are identical or exhibit statistically significant differences. In each repeat of this test, pairwise comparisons of species distributions are conducted and their localities pooled, their identities are then randomized and two new random samples are extracted to generate a set of pseudoreplicates. The results are compared with the true calculated niche overlap (see above). The lower the true niche overlap is in comparison to the scores created by the pseudoreplicates of the pooled samples, the more significant the niche difference between the two compared species. Finally, we classified species by altitudinal and habitat preference and compared all data.

## Results

### Molecular phylogenetics

**Figure 1:**
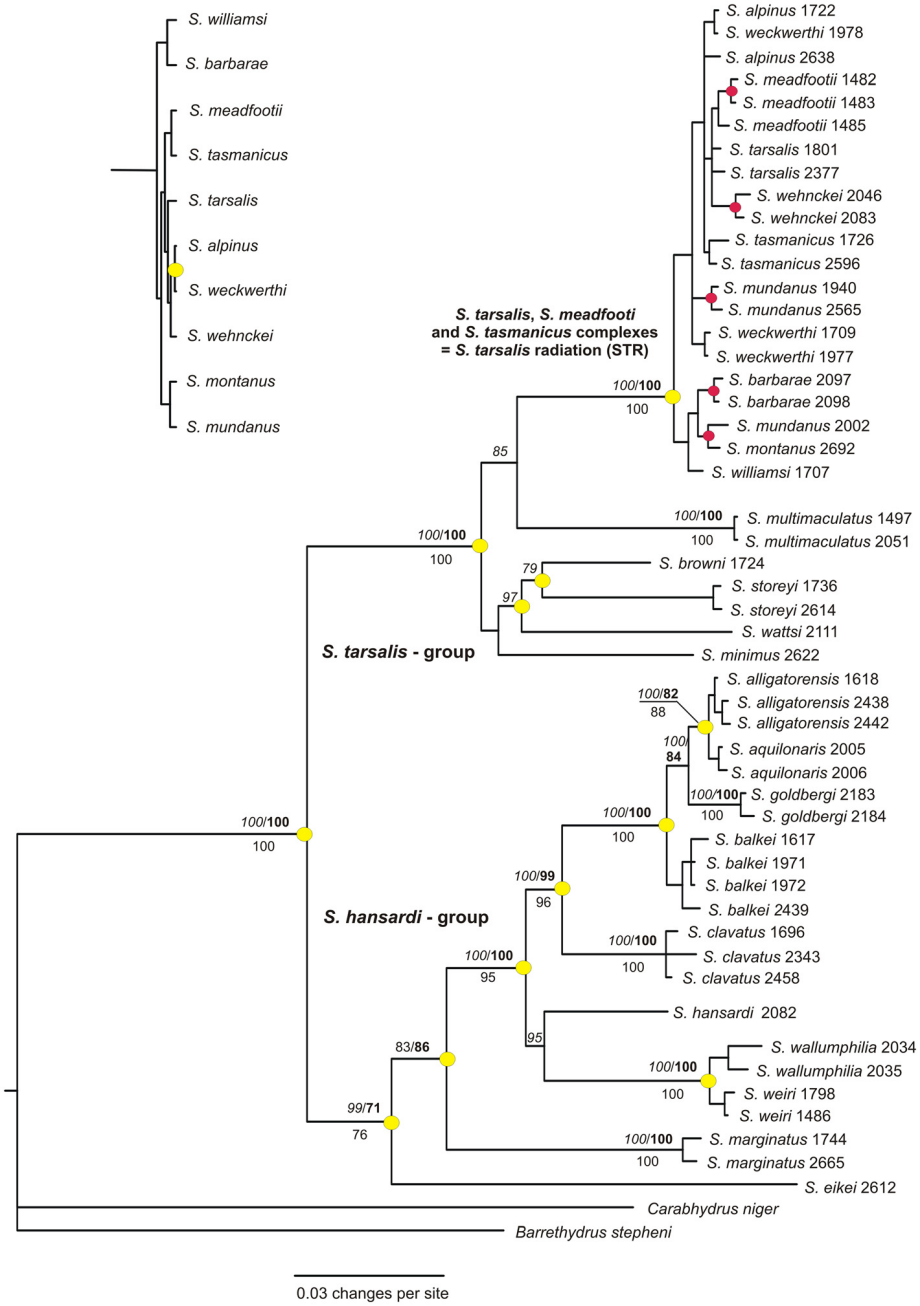
**Phylogram of the genus *Sternopriscus. *** The phylogram is based on a MrBayes tree with 7 gene loci and 3858 characters. Branch values are: MrBayes posterior probability (italic/above branch), RAxML bootstrap (bold/above branch), and TNT jackknife (below branch). Yellow circles mark nodes with *BEAST species tree posterior probabilities of 75 or more. Red circles mark nodes within the *S. tarsalis* radiation with PP, bootstrap and jackknife values of 75 or more (values not shown for layout reasons). Each tip represents one specimen. Specimen collection numbers are given after the species names. Upper left: *BEAST species tree fragment showing the *S. tarsalis* radiation specimens

Bayes factor analyses favored separate partitioning of genes and codon positions (17 partitions in total). This was the most complex partition strategy tested. Substitution models applied were according to jModeltest: the GTR + I + G model (16 S rRNA, mitochondrial non-protein-coding), the GTR + G model (*cox1*, *cob*, mitochrondrial protein-coding), the HKY + I + G model (18 S rRNA, nuclear non-protein-coding), and the HKY + G model (ARK, h3, h4, nuclear protein-coding). Bayesian, maximum likelihood, and maximum parsimony analyses revealed compatible topologies (Figure 1) that were largely congruent with the previously recognized classifications based on morphology. Here, we assign the four species previously supposed to be ‘phylogenetically isolated’ to either the *S. tarsalis* (*S. browni* and *S. wattsi*), or the *S. hansardii* (*S. eikei* and *S. marginatus*) group. Within the *S. tarsalis* group, all *S. tarsalis* complex species form a strongly supported clade (Figure 1).

The *BEAST species tree is largely congruent to the gene trees. The main difference is that in the gene trees, *S. multimaculatus* is the sister taxon to the STR, whereas in the *BEAST tree *S. minimus* is the sister taxon to the STR and *S. multimaculatus* is the sister taxon to all other members of the *S. tarsalis* group. Almost all species tree nodes within the STR are poorly supported. Notably, the analysis of the *BEAST run log file showed near-critically low posterior and prior effective sample sizes (< 120). This problem could neither be solved by repeating runs with higher sample frequencies nor with the application of simpler substitution models, as proposed in Pepper *et al.*[13], and indicates that the species tree results must be treated with caution.

The largest calculated *cox1 p*-distance between species in the STR was only 3.4% (*S. tarsalis*/*S. barbarae*), but interspecific distances may be as low as 0.3% (e.g., between *S. alpinus*, *S. mundanus* and *S. weckwerthi*, all belonging to different *S. tarsalis* complexes) or 0.2% (*S. alpinus/S. wehnckei*). Thus, no genetic distinction between the three complexes was possible because specimens often cluster with those belonging to other morphologically well-characterized species. This problem could not be solved by inspecting trees based on single or combined nuclear loci; the species *S. mundanus* and *S. weckwerthi* were polyphyletic in single-gene trees of *cob*, *cox1*, and ARK. The STR species shared identical haplotypes in all other nuclear genes studied.

### Diversification analyses

**Figure 2:**
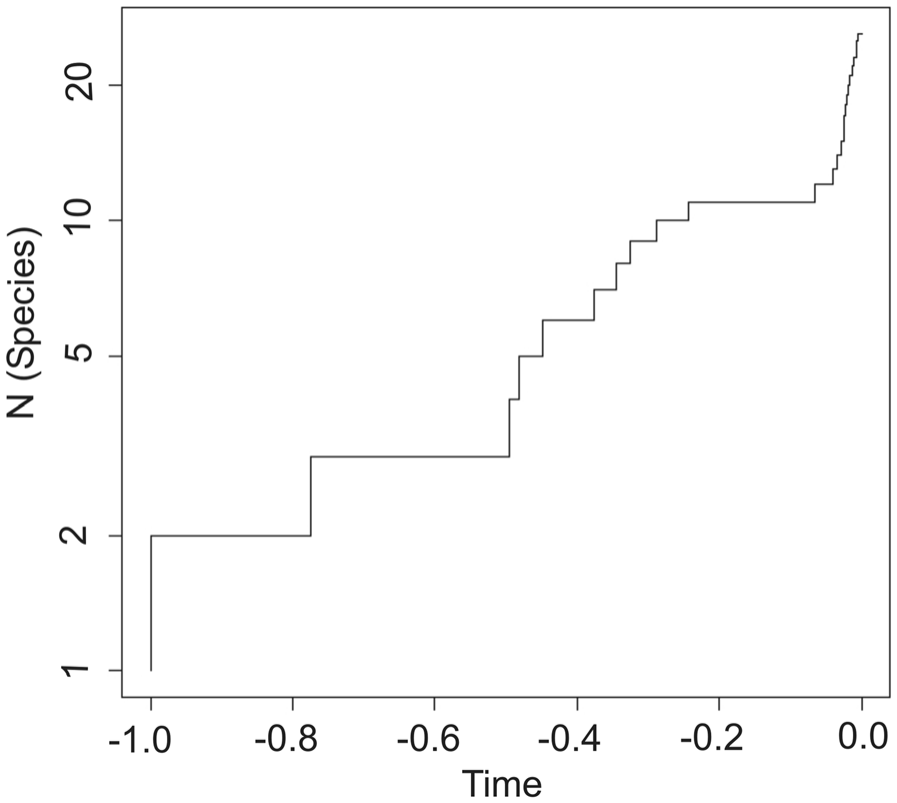
**Lineage-through-time (LTT) plot for the genus *Sternopriscus. *** Relative time (−1.0 is the time of the initial lineage split within the genus, 0.0 is the present) is given on the x-axis, number of species is given logarithmically on the y-axis

Figure 2 shows the LTT plot for *Sternopriscus*. APE yielded a positive γ value of 3.22 (p = 0.0013*). According to the mccr test, the critical value is 1.73 (p = 0.9*10E-3**) and is therefore met by the true value of γ. The test in Laser yielded a Yule-2-rate model as significantly better than the next best model, which was a constant rate birth-death model. The level of significance was highest (p = 0.0073*) for equal rates of b (birth) and d (death) (both 0.5), but all tested combinations of b and d yielded significant test results. In the test run in which the *S. tarsalis*-group was treated as a single clade, γ was negative but not significant at a value of −0.01 (p = 0.4956). This means that for this dataset the null hypothesis that the diversification rates have not decreased over time cannot be rejected.

The STR appears to have a thorough effect on the diversification analysis of the genus *Sternopriscus*. A high positive γ represents a rather unusual condition [6]. While many phylogenies are characterized by a decreasing rate of diversification (logistic growth or impact of extinctions [62]), a γ = 3.22 suggests a diversification rate that is highly increasing over time. This pattern is hard to explain in general. In the case of *Sternopriscus*, it appears appropriate to attribute this pattern to the recent speciation burst of the STR, which comprises 10 of 28 known species. This view is also supported by the test results that indicate a Yule-2-rate model as the most adequate, which fits to a sudden shift in diversification rates.

Papdopoulou *et al.*[44] suggested using substitution rates of 3.54% *cox1* divergence per MY which suggest an origin of the STR *c*. 0.96 MYA, and interspecific distances indicate divergence times as recent as 60,000 to 80,000 years ago. The slower substitution rate (2.3%) suggested by Brower [45] yields an approximate origin of the STR around 1.48 MYA and interspecific divergence times of 87,000 to 130,000 years ago (but see Papadopoulou *et al.*[44] for a discussion of these estimates). The equation by Mendelson & Shaw [16] was used to estimate speciation rates in the STR. Applying the proposed rate of Papdopoulou *et al.*[44], we estimate a speciation rate in the STR of 2.40 species per MY. Applying the proposed rate of Brower [45], we estimate a speciation rate in the STR of of 1.56 species per MY.

### Phylogeographic structure

**Figure 3:**
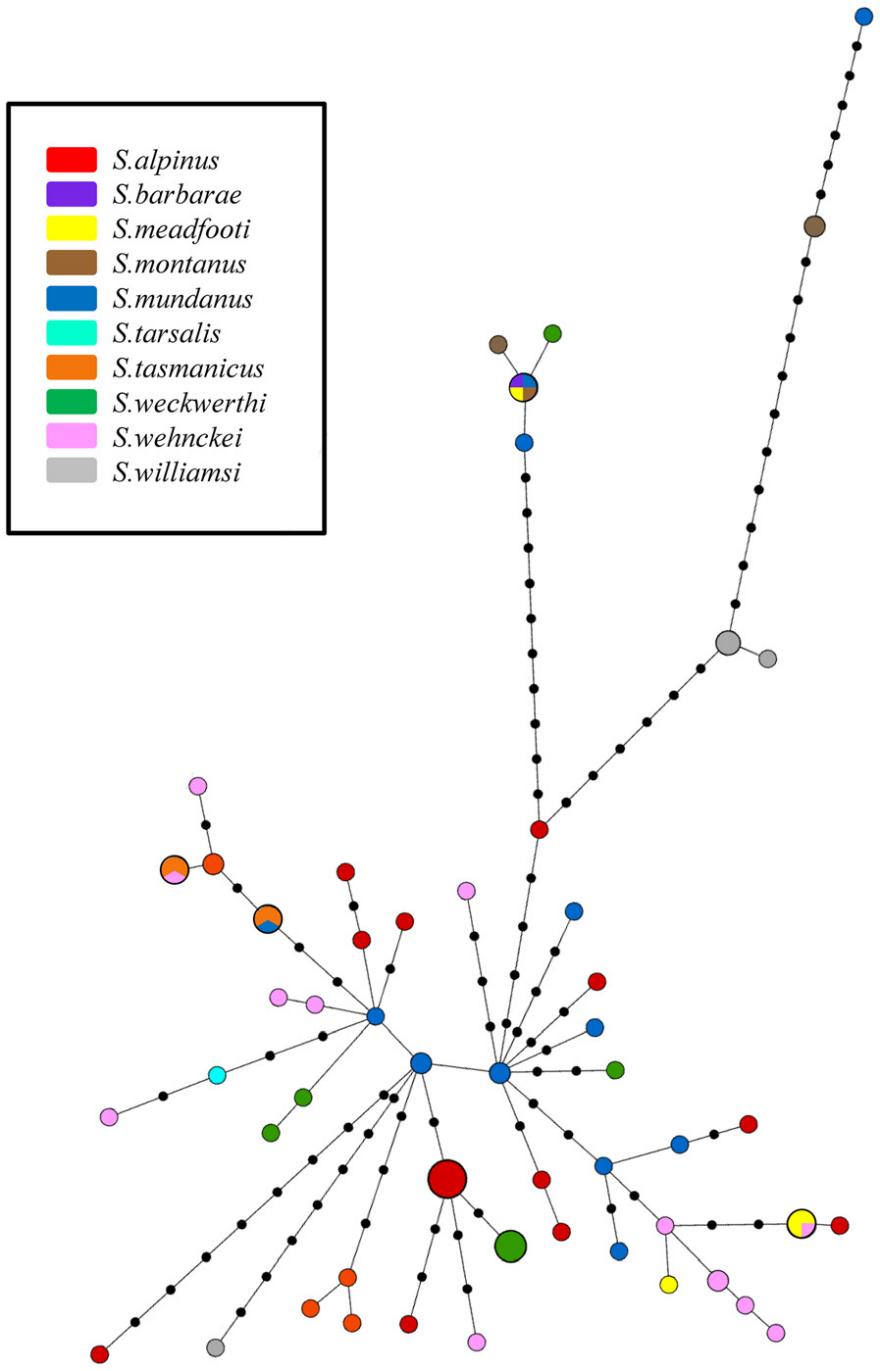
**Minimum spanning tree of haplotypes of the *Sternopriscus tarsalis *radiation.** The tree was created in Hapstar 0.5. Colors code the species determined according to morphology. Colored circles represent haplotypes, black dots represent mutational steps that are not represented by any haplotype

The matrix of 79 *cox1* sequences contained 69 polymorphic sites with a nucleotide diversity of π = 0.0121 and a haplotype diversity of H = 0.9815. We identified 61 distinct and mostly unique haplotypes within the STR with only 8 haplotypes comprising more than one sequence. Neither geographic nor taxonomic (Figure 3) mapping on the star-like MST yielded a comprehensive pattern. More precisely, no geographic structuring could be noticed based on the zoning of Australia, and the haplotypes of individuals of identical species were not systematically gathered in groups. Interestingly, the MST appears to be composed of two central haplotypes of South Australian and Victorian *S. mundanus* from which the rest of the sequences appears to have derived. In addition, even if there is a lack of geographical or taxonomic structuration, one might notice that several haplotypes representing different species are separated from the central network by a deep break of multiple mutation steps. While Tajima's D value does not significantly support a scenario of demographic expansion (D = −1.27773, p-value = 0.06), Fu's Fs significantly support such a demographic history (Fs = −35.731, p-value = 0.01) (see Tajima [47] and Fu [46] regarding the interpretation of Tajima's and Fu's statistics). However, the mismatch distribution analyses yield a multimodal distribution of the pairwise genetic distances, which favors a scenario of demographic equilibrium for the STR even if unimodal distributions are recovered only for recent and fast expansions [63]. 

### Incomplete lineage sorting vs. hybridization

*BEAST yielded a high relative locus rate of 2.332 for *cox1*, which was expected because many other markers included in our multilocus dataset, mainly nuclear markers, are known to evolve slower. The results of the JML run are given in Table 1. All species pairs exhibit genetic distances that are not significantly lower than expected. Thus, we cannot reject the hypothesis of incomplete lineage sorting in any cases.

**Table 1:** Results of the JML run

**Distance obs./exp.**	***S. alp.***	***S. bar.***	***S. mea.***	***S. mon.***	***S. mun.***	***S. tar.***	***S. tas.***	***S. wec.***	***S. weh.***	***S. wil.***
*S. alp.*		4.83	2.42	4.83	2.42	2.42	2.42	1.21#	1.21#	4.83
*S. bar.*	14.81		4.83#	2.42#	4.83#	4.83	4.83	4.83+	4.83	2.42
*S. mea.*	4.44	0		4.83#	2.42#	2.42	1.21	2.42+	2.42#	4.83
*S. mon.*	14.81	0	0		4.83#	4.83	4.83	4.83+	4.83	2.42
*S. mun.*	1.48	0	0	0		2.42	2.42#	2.42+	2.42#	4.83
*S. tar.*	5.92	23.70	8.89	23.70	4.44		2.42	2.42	2.42+	4.83
*S. tas.*	5.93	22.22	8.89	22.22	0	5.93		2.42	2.42#	4.83
*S. wec.*	0	1.48	1.48	1.48	1.48	5.93	4.44		1.21#	4.83
*S. weh.*	0	19.26	0	19.26	0	1.48	0	0		4.83
*S. wil.*	10.37	19.26	16.30	16.30	11.85	16.30	14.81	14.81	11.85	

Minimum genetic distance (*1,000), as estimated by JML, of STR species pairs. Lower left: observed minimum genetic distance. Upper right: expected minimum genetic distance (median). Species pairs in which the observed genetic distance is 0 due to the sharing of haplotypes are indicated by #. Species pairs in which the observed minimum genetic distance is higher than the expected distance are indicated by +. There is no case in which the probability that the minimum observed genetic distance is lower than expected is significant (p ≤ 0.05).

### Ecological niche modeling

**Figure 4:**
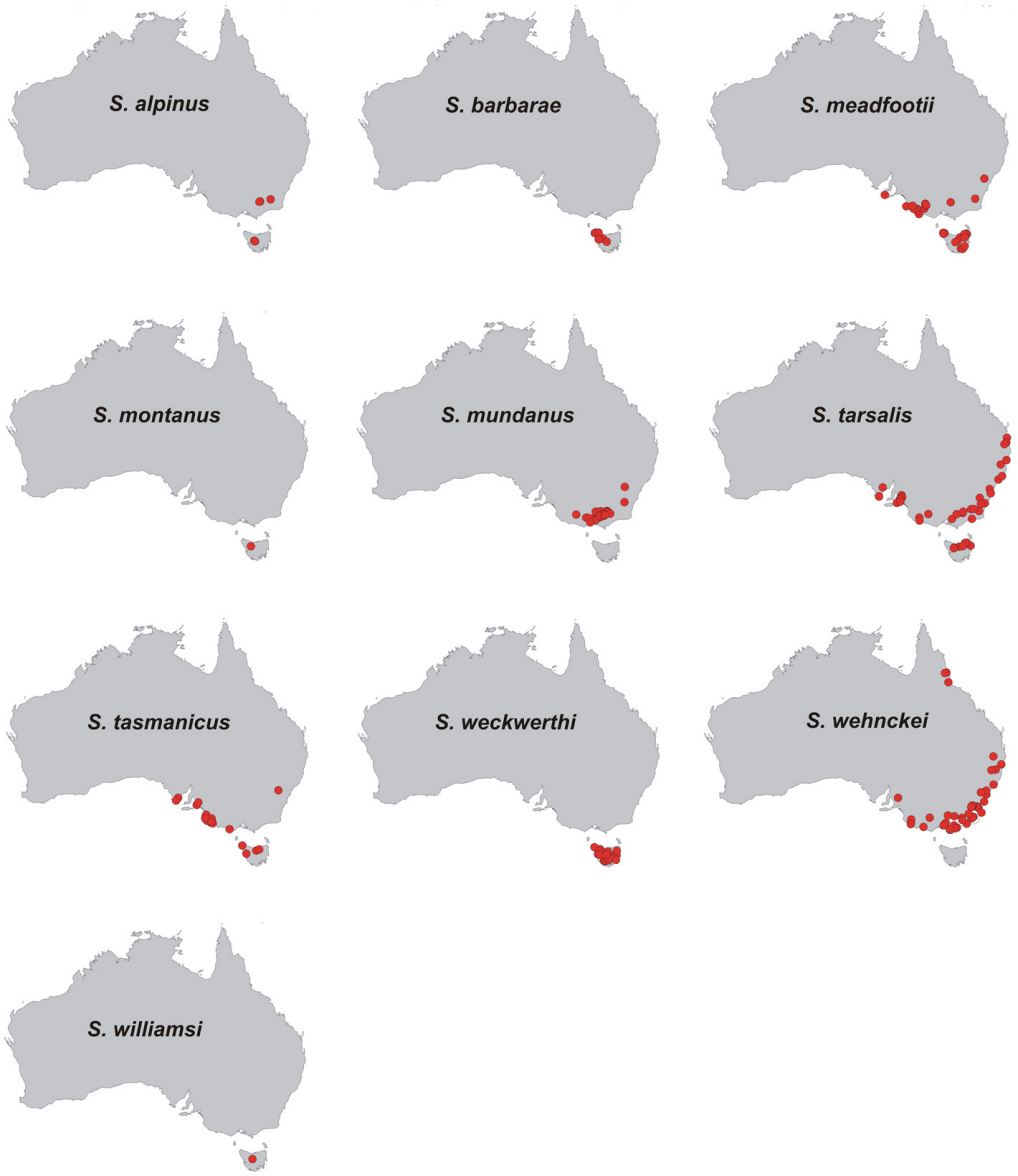
**Distribution of species of the *Sternopriscus tarsalis *radiation. ** Red dots represent specimen localities used for ecological niche modeling

**Figure 5:**
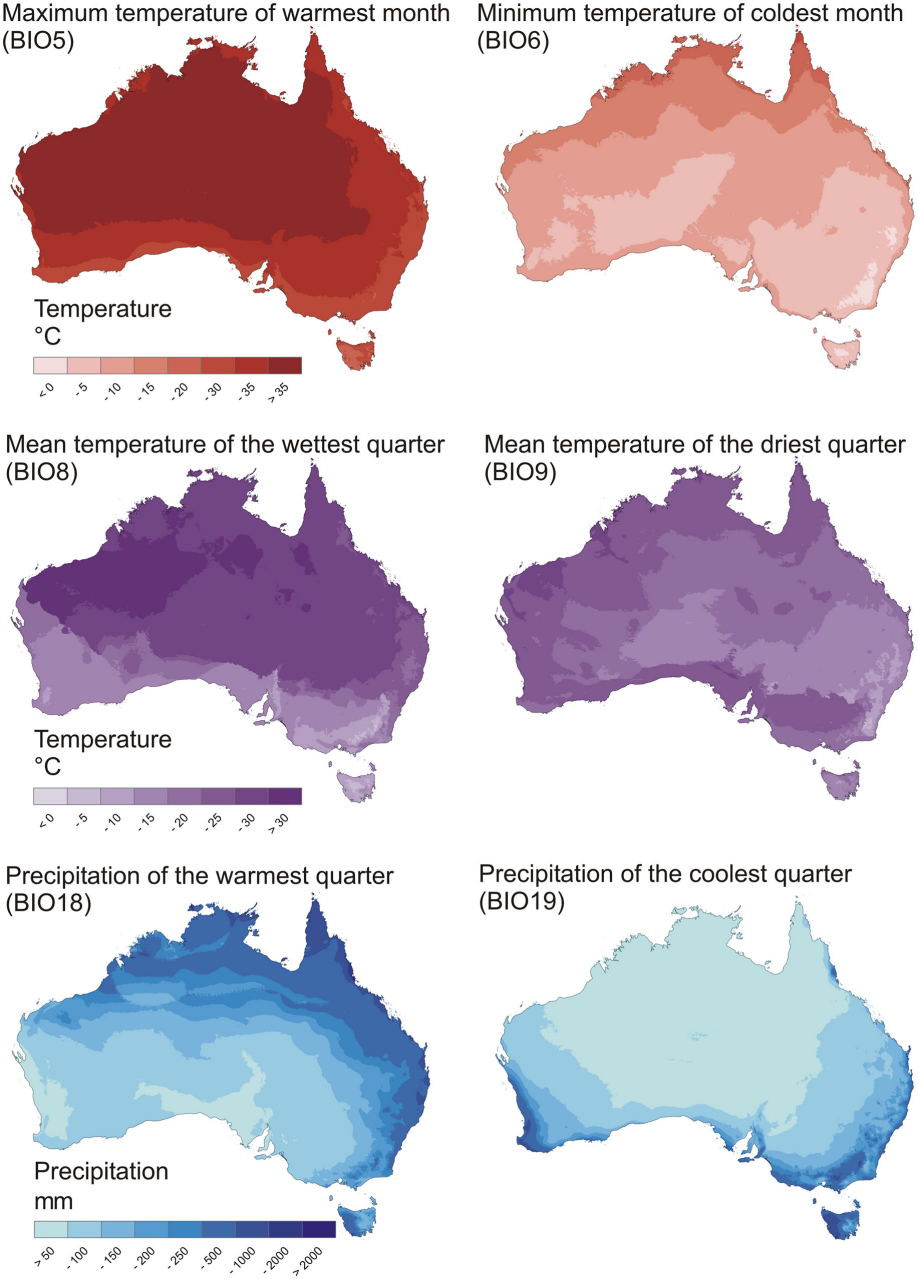
**Climate variables used for ENM creation.** Variables were selected to represent the effects of temperature, precipitation and seasonality

**Figure 6:**
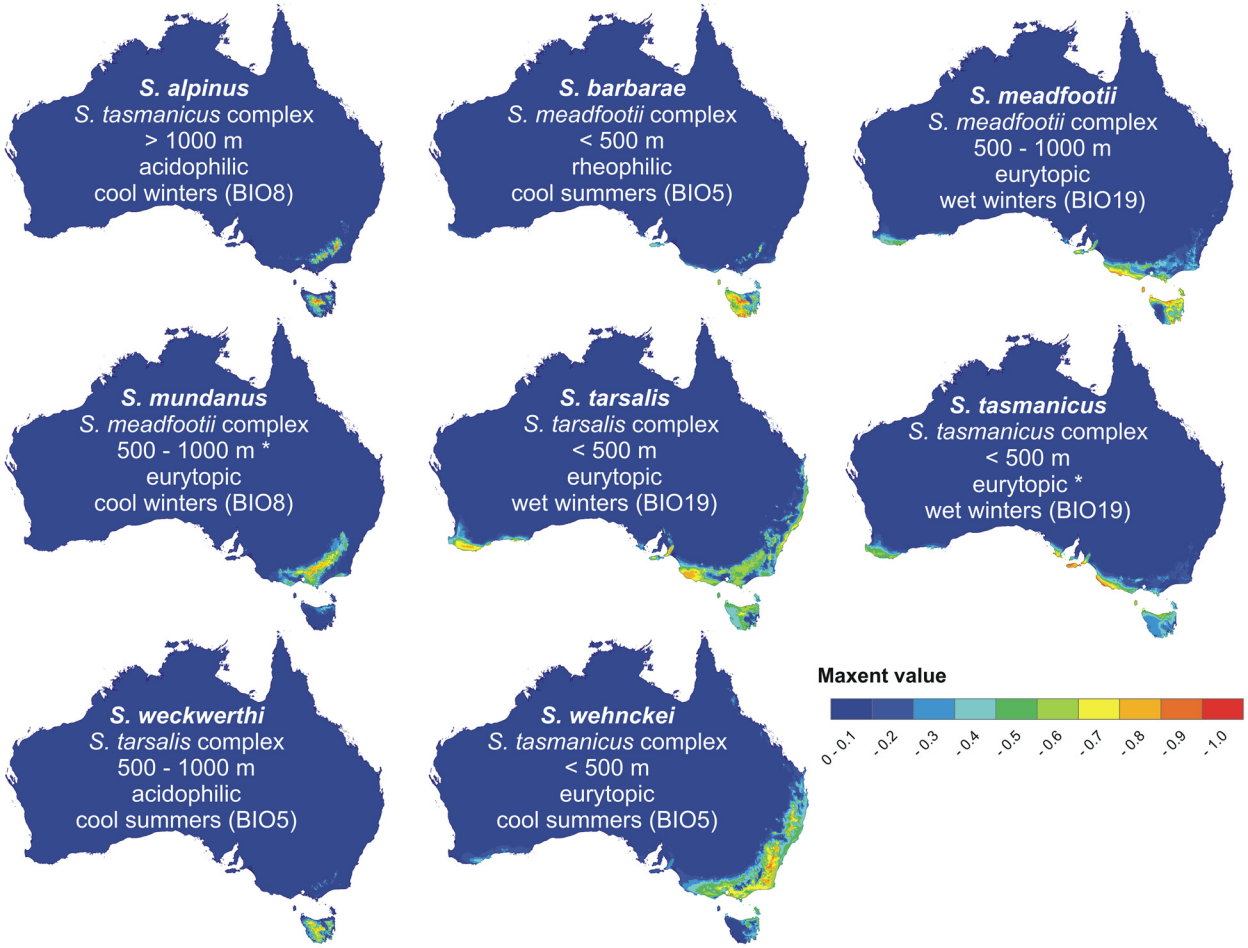
**Ecological Niche Models (ENMs) for species of the *Sternopriscus tarsalis *radiation. ** No ENMs were created for *S. montanus* and *S. williamsi* because of insufficient locality data. High Maxent values indicate high probabilities of occurrence of a species on a raster square (2.5 arc-minutes resolution). Maps include species name, taxonomic affinity, altitudinal range, habitat type and climate variable of highest importance in the ENM

Figure 4 summarizes all distribution points for all STR species and Figure 5 summarizes climate variables used for the creation of ENMs. The ENMs for the 8 STR species analyzed, supplemented with other ecological data, are given in Figure 6. AUC values for all models range from 0.981 to 0.997. Because all values are > 0.9, the ability to distinguish presence from random background points is considered "very good" for all models according to Swets [60]. We preliminarily selected the climate layers "maximum temperature of the warmest month" (BIO5), "minimum temperature of the coldest month" (BIO6), "mean temperature of the wettest quarter" (BIO8), "mean temperature of the driest quarter" (BIO9), "precipitation of the wettest month" (BIO13), "precipitation of the driest month" (BIO14), "precipitation of the warmest quarter" (BIO18) and "precipitation of the coldest quarter" (BIO19). In our final selection, we omitted BIO13 and BIO14 because of correlation coefficients with other variables of r² > 0.75. Thus, all models presented here are based on six climate variables. Jackknifing to measure the importance of variables showed that either "maximum temperature of the warmest month" (BIO5: *S. barbarae*, *S. weckwerthi*, *S. wehnckei*), "mean temperature of the wettest quarter" (BIO8: *S. alpinus*, *S. mundanus*), or "precipitation of the coldest quarter" (BIO19: *S. meadfootii*, *S. tarsalis*, *S. tasmanicus*) were the most important variables in creating ENMs. Niche overlap values (I and D) and identity test results are given in Table 2. The results of the identity test are highly significant (Bonferroni corrected) for I in all and for D in nearly all pairwise species comparisons. However, the null hypothesis of identity in the ENMs of two compared species can be rejected only if the true calculated niche overlap is below the 99.9% confidence interval of the values generated in the identity test. In a few cases, the true calculated niche overlap is above this interval, and the null hypothesis of niche identity cannot be rejected [61]. 

**Table 2:** Results of the niche identity test

**Overlap D/I**	***S. alp.***	***S. bar.***	***S. mea.***	***S. mun.***	***S. tar.***	***S. tas.***	***S. wec.***	***S. weh.***
*S. alp.*	0	0.674**	0.682**	0.676**	0.661*#	0.651**#	0.648**	0.571**
*S. bar.*	0.506**	0	0.733**#	0.569**	0.680**#	0.735**#	0.755**	0.582**
*S. mea.*	0.481**	0.589**#	0	0.691**	0.801**	0.847**#	0.606**	0.684**
*S. mun.*	0.474**	0.327**	0.496**	0	0.661**	0.602**	0.520**	0.637**
*S. tar.*	0.476	0.472	0.711**	0.456**	0	0.759**	0.548**	0.756**
*S. tas.*	0.433**	0.560	0.762**#	0.378**	0.648**	0	0.583**	0.627**
*S. wec.*	0.451**	0.642**	0.367**	0.241**	0.282**	0.331**	0	0.459**
*S. weh.*	0.374**	0.356**	0.523**	0.444**	0.639**	0.419**	0.177**	0

Niche overlap values (D and I), calculated with ENMtools, are given for species pairs and are mostly lower than the randomized overlap levels generated in the identity test at significant (*, p ≤ 0.05, Bonferroni corrected) or highly significant (**, p ≤ 0.001, Bonferroni corrected) level. This means that niches are more divergent than expected at random. In some cases, results are not significant, or significantly higher than the randomized overlap (indicated by #). In these cases, niches are not more divergent than expected by random. Note that results yielded by D and I do not accord in all cases.

### Ecological analyses

All species of the STR were compared for their preferences in altitude and habitat and for the most important climate factor in their ENM, which resulted from the jackknifing test in the ENM runs. Table 3 displays these three factors for all species coded by numbers for easy comparison. Only *S. tasmanicus* and *S. tarsalis* are identical in all three factors. *S. montanus* and *S. williamsi* might be identical to *S. alpinus* or *S. weckwerthi* depending on the most important climate factor, but no ENMs could be created. Within each of the three complexes in the *S. tarsalis* group, no two species are identical in all three factors.

**Table 3:** **Taxonomic affinities and ecological preferences of species in the *Sternopriscus tarsalis *radiation**

**Species**	**Complex**	**Altitude**	**Habitat**	**Climate**
*S. alpinus*	*2*	2	2	1
*S*. *tasmanicus*	*2*	0	1*	2
*S. wehnckei*	*2*	0	1	0
*S. barbarae*	*1*	0	0	0
*S. meadfootii*	*1*	1	1	2
*S. montanus*	*1*	2	2	?
*S. mundanus*	*1*	1 **	2	1
*S. tarsalis*	*0*	0	1	2
*S. weckwerthi*	*0*	2	2	0
*S. williamsi*	*1*	1	2	?

Complex: 0 = *S. tarsalis*, 1 = *S. meadfootii*, 2 = *S. tasmanicus*. Altitude: preferred altitude range, 0 = < 500 m, 1 = 500 – 1000 m, 2 = > 1000 m. Habitat: 0 = rheophilic, 1 = eurytopic, 2 = acidophilic. Climate: according to the dominating climate variables in the ENM, 0 = cool summers, 1 = cool winters, 2 = wet winters. *: Also occurs in habitats with moderate salinity. **: Actual altitudinal range is 200 – 1550 m.

## Discussion

In the opening section of this article, we suggested three hypotheses: (1) species delimitation in the STR can be supported by genetic or ecological data; (2) the STR species originated in a rapid Pleistocene diversification event; and (3) Pleistocene climate oscillations promoted the radiation of the STR. In the following, we will discuss how our results support these hypotheses.

Our data shows that the molecular methods applied in our study do not serve to unambiguously distinguish and delimit the species of the STR. This is because of the widespread genotype sharing of mitochondrial genes and lack of diversification in nuclear genes between these species. However, the analysis of our ecological data shows that STR species appear to respond differently to ecological variables. Below, we initially discuss whether incomplete lineage sorting or hybridization may have caused the abundance of shared haplotypes in the STR. Then, we discuss the importance of the results of our ecological analyses in the context of the entire genus, and specifically for the STR.

Genotype sharings between species may be explained by incomplete lineage sorting, by hybridization, or a combination of both. Funk & Omland [19] also mention imperfect taxonomy, inadequate phylogenetic information and paralogs as causes for genotype sharing. However, the taxonomy of *Sternopriscus* based on morphological characters is well supported [27,28], and our multi-gene phylogeny is well supported by different analytical approaches. Paralogs can almost certainly be excluded because the patterns of species polyphyly are repeated by different mitochondrial and nuclear markers.

Hybridization, as a reason for genotype sharing in closely related species, has been proposed for various animal groups [64,65], including groups with strong sexual selection (e.g., mating calls [66]), and has been shown to contribute to speciation [64]. However, in the case of *Sternopriscus*, the results of our analyses, the diversity in genital morphology, and the absence of specimens identifiable as hybrids, do not support hybridization [67]. Incomplete lineage sorting, or the retention of ancestral polymorphism, is the more likely explanation for genotype sharing in the case of the STR. Incomplete lineage sorting has often been recognized as a problem in resolving phylogenies of young and closely related taxa [68]. This phenomenon affects nuclear loci more commonly than faster evolving mitochondrial loci, but mitochondrial genes can be equally affected, particularly in closely related taxa where hardly any diversification in nuclear genes is found [19]. Incomplete lineage sorting as an explanation for haplotype sharings in the STR supports the view that the STR is a recent radiation.

A comparison of our ecological findings concerning the STR species with data on other *Sternopriscus* species shows that the STR occupies ecological ranges similar to those of other related species. The currently known altitudinal distribution and ecology of all *Sternopriscus* species in Australia is shown in Additional file 3: Table S3, modified after Hendrich & Watts [27,28]. 10 species of the genus are rheophilic and inhabit rivers and streams that are mainly of intermittent character. 11 species are acidophilic and live in seasonal or permanent swamps, ponds and pools of different types of peatlands. 7 species appear to be more or less eurytopic and occur in various water bodies in open or forested country. The highest species diversity is in lowland or coastal areas and hilly or low mountain ranges from 0 to 500 m. Only 6 species were collected at 1000 m or above (*S*. *alpinus*, *S*. *meadfootii*, *S*. *montanus*, *S*. *mundanus*, *S. williamsi* and *S*. *weckwerthi*).

Within the STR, all species inhabit broadly overlapping areas in mesic southeast Australia, except for a few localities of *S. wehnckei* in the northeast (the Eastern Coastal Australia region and small parts of the Murray-Darling region of Abell *et al.*[29]. Many species also inhabit Tasmania, including two endemics (Bass Strait Drainages and Southern Tasmania). ENMs indicate niche diversification within this group of closely related and broadly sympatric species. Aside from the high levels of significance in the identity test, the degree of niche diversification is hard to measure. Therefore, we rely on the importance of the various climate variables used to characterize the species ENMs. The variables of highest importance are "maximum temperature of the warmest month" (BIO5), "mean temperature of the wettest quarter" (BIO8), or "precipitation of the coldest quarter" (BIO19). Figure 5 shows that all the species studied inhabit areas with relatively low maximum temperatures, with the lowest on Tasmania. The two species most characterized by this factor are the two Tasmanian endemics, *S. barbarae* and *S. weckwerthi*. A distinction between the two remaining factors is more difficult. Considering Figure 5, "mean temperature of the wettest quarter" is lowest in areas where winters (the wettest quarter in our region of interest) are cold, whereas "precipitation of the coldest quarter" is highest where winters are wet. Some species (e.g., the high-altitude *S. alpinus*) may be tolerant of winter temperatures that are too low for other species, whereas other species are more dependent on sufficient precipitation. Species that require the latter are eurytopic species that also inhabit ephemeral waters, such as ponds at the edge of rivers and creeks, which are only filled after heavy rainfall. The acidophilic species, which inhabit more permanent water bodies with dense vegetation, are often "cold winter" species.

The low divergences between haplotypes in the STR species suggest that these species originated in a recent and rapid radiation. Unfortunately, we could not rely on any calibration points to support our molecular clock approach. Instead, we attempted to estimate the origin of the STR based on standard *cox1* mutation rates [44,45]. We estimated an origin of *c.* 0.96 to 1.48 MYA, which leads to an estimated speciation rate of 2.40 or 1.56 species per MY. Genetic distance might indicate the age of the ancestral species, however divergence time estimates for the extant species should not be considered reliable beyond assumption of a comparably recent origin of the STR. This fact alone, however, suggests that the STR is an exceptional event for what is known of aquatic beetles. For other insect groups, little evidence exists for similarly fast diversification events. The fastest rate (4.17 species per MY) was estimated for a clade of 6 species of Hawaiian crickets over 0.43 MY [16]. However, in the same study, for a related clade comprising 11 species, the estimated rate was much lower at 1.26 species per MY over 1.9 MY. Additional estimates are available for *Galagete* moths in the Galapagos [17] of 0.8 species per MY (n = 12, t = 1.8 MY) and for Japanese *Ohomopertus* ground beetles [18] of 1.92 (n = 15, t = 1.4 MY) to 2.37 species per MY (n = 6, t = 0.76 MY). The average speciation rate in insects was proposed to be 0.16 species per MY [15]. This comparison shows that rapid radiation events, as exemplified in the STR, appear to be exceptional among insects and particularly in continental faunas because all other examples recorded were island radiations.

Species groups that originated from rapid radiation events have been detected in almost all organismic groups and habitats [69]. An overview of many recent and past events suggests three major promoters of rapid radiations: the appearance of a key innovation that allows the exploitation of previously unexploited resources or habitats [70], the availability of new resources [71], and the availability of new habitats, e.g., because of a rare colonization event or drastic environmental changes [72,73]. In the case of the STR, we find no evident key innovation distinguishing this group from other *Sternopriscus* species. We have no data concerning internal morphology or physiology. Additionally, our data show that the observation that STR species have ecological requirements similar to those of other *Sternopriscus* species does not indicate the presence of any key innovations. There is also no indication of any new resource that could be specifically exploited by the STR species. Therefore, we explore the possibility that drastic environmental changes during the Pleistocene climate oscillations mediated the radiation of the STR species.

During most of the Cenozoic, the climate of Australia was hot and humid and currently remains so in the northern rainforest areas [11]. Aridification began in the Miocene (*c*. 15 MYA) and gradually led to the disappearance of forests and to the spread of deserts over much of the present continent. Most of today's sand deserts, however, are geologically younger and appeared only after the final boost of aridification that accompanied the Ice Ages, particularly since the later Pleistocene (*c*. 0.9 MYA). The climate was subjected to large oscillations in temperature and rainfall, which drove many groups of organisms into refugia and also promoted speciation [12,13]. Our results also document a strong and abrupt increase in speciation in the genus *Sternopriscus* about 1 to 1.5 MYA, represented by the STR. This age estimate is congruent with the Pleistocene oscillations. Byrne *et al.*[12] present cases of organisms restricted to mesic habitats that were formerly most likely more widespread, but today occupy relictual areas with suitable climates. However, some of the young species of the STR occupy rather large areas in southwestern Australia. This distribution indicates good dispersal abilities, which are necessary for organisms that inhabit habitats of relatively low persistence [74]. Ribera & Vogler [75] argue that for this reason, beetle species that inhabit lentic aquatic habitats often have better dispersal abilities than those inhabiting lotic habitats. However, it is possible that the STR species of lotic habitats only recently derived from an ancestor adapted to lentic habitats with good dispersal abilities that are maintained in the newly derived species.

Speciation in Pleistocene refugia was previously described for dytiscid beetles on the Iberian Peninsula [9]. During the Pleistocene climate oscillations, the ancestral species of the STR might have been forced into ongoing cycles of retreating into, and the re-expansion from, refugia. Under the recurrent, extremely unsuitable climate conditions, the isolation of small populations over many generations might have promoted speciation and the fixation of morphological traits. This scenario might also explain the lack of clear geographic or taxonomic structuring in the striking haplotypic diversity presented by the STR species. This diversity might be attributed to the cycles of expansion and retreat that repeatedly isolated haplotypes in various geographic locations before newly allowing the expansion and colonization of other areas.

The phenomenon of groups of young and closely related species within a defined distributional range is most familiar in ichthyology, in which it was termed "species flock". Among the most prominent species flocks are the cichlids of the African Great Lakes and other lake ecosystems around the world, the Sailfin Silversides of Sulawesi, and the Notothenioid Antarctic Ice Fishes (see review in Schön & Martens [76]). Schön & Martens [76] summarize the criteria for naming a group of species a species flock as "speciosity [= species-richness], monophyly and endemicity". Compared with the large fish species flocks, the STR is poor in species. Nevertheless, the number of species is "disproportionally high" [77] in relation to the surrounding areas, as no other region in Australia is inhabited by a comparable assemblage of closely related species. In the last decade, an increasing number of less species-rich radiations have been termed species flocks with as little as 3 or 4 species [76,78]. Most other species flocks inhabit lakes, islands or archipelagoes. These are areas more "narrowly circumscribed" [77] than the area of endemism of the STR, which can be broadly termed "the southeast Australian region". Most STR species have relatively large ranges that do not share a common limit and sometimes do not even overlap. Our results show that STR species often occupy different habitat types. Additionally, the clade is not strictly endemic to southeastern Australia, as shown by the northeastern records of *S. wehnckei*. Based on this criterion, other rapid radiations among insects [16,17] are much more adequate examples of species flocks.

## Conclusions

Our results provide evidence that STR species are the result of an extremely recent, most likely Pleistocene, radiation. The STR species cannot be distinguished with the molecular methods used in this study, however, the species show clear divergences in their responses to ecological factors of habitat type and climate. We proposed a scenario in which the Pleistocene climate oscillations led to the repeated restriction and expansion of the ranges of the ancestral species of the STR, which may have promoted fixation of ecological adaptations and morphological traits in small and isolated populations restricted to refugia. This suggests that *Sternopriscus* is an example for the hypothesis that Pleistocene refugia promoted speciation.

Taking this scenario into account, the STR does not appear as an evolving or fully evolved species flock but as a radiation based on a species flock. While possibly confined to a narrowly circumscribed area during the Pleistocene, the STR species were able to break the boundaries of their refugia with the end of the Ice Ages and increase their ranges. Today, because the species are no longer confined to a common limited area, the term "species flock" may best fit a stage in speciation the STR has previously passed.

## Abbreviations

ENM: Ecological niche modeling; MST: Minimum spanning tree; MY: Million years; MYA: Million years ago; STR: *Sternopriscus tarsalis* radiation.

## Competing interests

The authors declare that they have no competing interests.

## Authors' contributions

OH performed the laboratory work, the molecular genetic studies, the diversification analyses, the ecological niche modeling and analyses, and drafted the manuscript. LH collected the samples and ecological data and helped to draft the manuscript. ME coordinated the diversification analyses. EFAT conducted the phylogeographic analyses. MJG conducted the analysis of hybridization vs. incomplete lineage sorting. MB conceived the study, participated in its design and coordination, and helped to draft the manuscript. All authors read and approved the final manuscript.

## Supplementary Material

Additional file 1 **Table S1.** Sequences of primers used for PCR and sequencing. Forward (F) and reverse (R) primers are given. Mitochrondrial gene loci: CO1 = cytochrome C oxidase 1, CytB = cytochrome B oxidase, 16 S = 16 S ribosomal RNA. Nuclear gene loci: H3 = histone 3, H4 = histone 4, ARK = arginine kinase,18 S = 18 S ribosomal RNA. I = inosine. Click here for file

Additional file 2 **Table S2.** Localities of *Sternopriscus* species used in Ecological Niche Modeling. Coordinates are given in decimal degrees. Click here for file

Additional file 3 **Table S3.** Ecological data on all *Sternopriscus* species. Data from Hendrich & Watts [27,28]. Click here for file
