# Supplementary material for: Pleistocene climate change promoted rapid diversification of aquatic invertebrates in Southeast Australia
Source: BMC Evol Biol. 2012 Aug 9;12:142. doi: 10.1186/1471-2148-12-142 (PMC3503846; doi:10.1186/1471-2148-12-142)
Supplement: Additional file 1 — Table S1. Sequences of primers used for PCR and sequencing. Forward (F) and reverse (R) primers are given. Mitochrondrial gene loci: CO1 = cytochrome C oxidase 1, CytB = cytochrome B oxidase, 16 S = 16 S ribosomal RNA. Nuclear gene loci: H3 = histone 3, H4 = histone 4, ARK = arginine kinase,18 S = 18 S ribosomal RNA. I = inosine. [file 1471-2148-12-142-S1.pdf]

Table S1: Sequences of primers used for PCR and sequencing.

Forward (F) and reverse (R) primers are given. Mitochrondrial gene loci: CO1 = cytochrome C oxidase 1, CytB = cytochrome B oxidase, 16S = 16S ribosomal RNA. Nuclear gene loci: H3 = histon 3, H4 = histon 4, ARK = arginine kinase,18S = 18S ribosomal RNA. I = inosine.

**Gene**	**Primer**	**F/R**	**Primer sequence**	**Reference**
CO1	Jerry	F	CAA CAT TTA TTT TGA TTT TTT GG	Simon et al. 1994
	Pat	R	TCC AAT GCA CTA ATC TGC CAT ATT A	
CytB	CB3	F	GAG GAG CAA CTG TAA TTA CTA A	Baraclough et al. 1999
	CB4	R	AAA AGA AA(AG) TAT CAT TCA GGT TGA AT	
16S	M14	F	CGC CTG TTT AAC AAA AAC AT	Simon et al. 1994
	M223	R	GGT CCC TTA CGA ATT TGA ATA TAT CCT	
H3	H3aF	F	ATG GCT CGT ACC AAG CAG AC(AG) CGC	Colgan et al. 1998
	H3aR	R	ATA TCC TT(AG) GGC AT(AG) AT(AG) GTG AC	
H4	H4F2s	F	T(CG)C GIG A(CT)A ACA T(CT)C AGG GIA TCA C	Pineau et al. 2004
	H4F2er	R	C(GT)(CT) TTI AGI GC(AG) TAI ACC AC(AG) TCC AT	
ARK	AK183F	F	GAT TCT GGA GTC GG(AGCT) AT(CT) TA(CT) GC(AGCT) CC(CT) GA(CT) GC	Wild & Maddison 2008
	AK939R	R	GCC (AGCT)CC (CT)TC (AG)GC (CT)TC (AG)GT GTG (CT)TC	
18S	18S 5'	F	GAC AAC CTG GTT GAT CCT GCC AGT (1)	Shull et al. 2001
	18S b5.0	R	TAA CCG CAA CAA CTT TAA T (1)	

Baraclough, T.G., Hogan, J.E., & Vogler, A.P. 1999 Testing whether ecological factors promote cladogenesis in a group of tiger beetles (Coleoptera: Cicindelidae). Proc. R. Soc. Lond. B. 266, 1061-1067.

Colgan, D.J., McLauchlan, A., Wilson, G.D.F., Livingston, S.P., Edgecombe, G.D., Cassis, G. & Gray, M.R. 1998 Histone H3 and U2 snRNA DNA sequences and arthropod molecular evolution. Aust. J. Zool. 46, 419-437.

Pineau, P., Henry, M., Suspène, R., Marchio, A., Dettai, A., Debruyne, R., Petit, T., Lécu, A., Moisson, P., Dejean, A., et al. 2004 A Universal Primer Set for PCR Amplification of Nuclear Histone H4 Genes from All Animal Species. Mol. Biol. Evol. 22, 582-588.

Shull, V.L., Vogler, A.P., Baker, M.D., Maddison, D.R. & Hammond, P.M. 2001 Sequence Alignment of 18S Ribosomal RNA and the Basal Relationships of Adephagan Beetles: Evidence for Monophyly of Aquatic Families and the Placement of Trachypachidae. Syst. Biol. 50, 945-969.

Simon, C., Frati, F., Beckenbach, A.T., Crespi, B., Liu, H. & Flook, P. 1994 Evolution, weighting, and phylogenetic utility of mitochondrial gene sequences and a compilation of conserved polymerase chain reaction primers. Ann. Entomol Soc. Am. 87, 651-701.

Wild, A.L. & Maddison, D.R. 2008 Evaluating nuclear protein-coding genes for phylogenetic utility in beetles. Mol. Phylogenet. Evol. 48, 877-891.
