# Supplementary material for: Pleistocene climate change promoted rapid diversification of aquatic invertebrates in Southeast Australia
Source: BMC Evol Biol. 2012 Aug 9;12:142. doi: 10.1186/1471-2148-12-142 (PMC3503846; doi:10.1186/1471-2148-12-142)
Supplement: Additional file 3 — Table S3. Ecological data on all Sternopriscus species. Data from Hendrich & Watts [27,28]. [file 1471-2148-12-142-S3.pdf]

Table S3: Ecological data on all *Sternopriscus* species.

Data from Hendrich & Watts [28,29].

**Species**	**Altitude**	**Habitat**
S. alpinus	Above 1000 m	Acidophilic
S. alligatorensis	50 - 100 m	Rheophilic
S. aquilonaris	50 - 100 m	Rheophilic
S. balkei	50 - 100 m	Rheophilic
S. barbarae	50 - 300 m	Rheophilic
S. browni	0 - 400 m	Eurytopic
S. clavatus	20 - 700 m	Eurytopic
S. eikei	20 m	Acidophilic
S. emmae	50 – 100 m	Rheophilic
S. goldbergi	50 - 100 m	Rheophilic
S. hansardii	200 - 500 m	Rheophilic
S. meadfootii	200 - 1000 m	Eurytopic
S. marginatus	50 - 400 m	Rheophilic
S. minimus	0 - 100 m	Acidophilic
S. mouchampsi	0 - 100 m	Acidophilic
S. montanus	Above 1000 m	Acidophilic/rheophilic
S. multimaculatus	0 - 100 m	Eurytopic
S. mundanus	200 - 1550 m	Acidophilic
S. pilbaraensis	50 - 500 m	Rheophilic
S. storeyi	0 - 50 m	Acidophilic
S. tarsalis	0 - 500 m	Eurytopic
S. tasmanicus	0 - 500 m	Eurytopic
S. wallumphilia	0 - 200 m	Acidophilic
S. wattsi	0 - 100 m	Eurytopic
S. weckwerthi	200 - 1000 m	Acidophilic
S. wehnckei	0 - 500 m	Eurytopic
S. weiri	0 - 50 m	Acidophilic
S. williamsi	Above 1000 m	Acidophilic
